# From fork to farm, locally: social acceptance pathways for human excreta-derived fertilisers across three European regions

**DOI:** 10.1007/s42532-025-00236-x

**Published:** 2025-12-09

**Authors:** Viktor Varjú

**Affiliations:** 1 Institute for Regional Studies, Pécs, Hungary, Transdanubian Research Department, ELTE CERS, Budapest, Hungary; 2 Institute for Rural Development and Sustainable Economy, Magyar Agrár- és Élettudományi Egyetem (MATE), 40. Guba S. str., Kaposvár, 7400 Hungary; 3 University of Pécs, Faculty of Humanities and Social Sciences, Department of Community and Social Studies, 2. Rókus str., Pécs, 7624 Hungary

**Keywords:** Social acceptance, Human excreta based fertiliser, NIMBY, Trust, Farm to fork to farm, Germany, Spain, Sweden, Regional difference, Circular transition

## Abstract

This research was carried out to better understand the attitudes of everyday people and stakeholders towards the new innovation of bio-based fertilisers made from human excreta. This research used the circular influencing model (CIM) as an analytical framework to understand the social acceptance of fertilisers made from urine and faecal matter. To do so, the model was tested in the three pilot regions of the P2GreeN Horizon Europe project, in Gotland (Sweden), in the North German Plain region and in the Axarquia region (Spain). In these regions, we used a survey questionnaire for everyday people as well as focus group and semistructured interviews with stakeholders. This qualitative analysis-based paper extends the literature on testing influencing models that examine acceptance at both the general and spatial levels. Additionally, it provides crucial insights for policymakers in understanding the barriers to the social acceptability of human-origin bio-based fertilisers. The results show that Swedish society leads the way in adopting this new solution. It was also clearly revealed that local municipality policymakers are best placed to promote adoption through local channels.

**Supplementary Information:**

The online version contains supplementary material available at 10.1007/s42532-025-00236-x.

## Introduction

The circular economy (CE) is now a widely researched concept, not only from the perspectives of innovation and business models but also from a social science approach, as many social conditions must be met for the circular transition (Varjú 2020, p. 5). The concept of the CE focuses on closing material loops to achieve ‘zero waste’ and improve the security of supply while simultaneously reducing environmental impacts (Azizli 2021, p. 36; Kalmykova et al. 2018, p. 190; Varjú and Óvári 2024, p. 1). Challenges across time and space are often displaced and require a complex approach that takes into consideration socio-spatial context (Amenta et al. 2019, p. 5; Calisto Friant et al. 2020; Kirchherr et al. 2017, p. 221; Mezei and Varjú 2019, p. 58; Song et al. 2019, p. 55). Local municipalities and stakeholders need a collaborative environment that shifts local society towards circularity (Ordonez and Martins 2023, p. 30; Remøy et al. 2019, p. 1; Varjú and Óvári 2024, p. 1), and this environment should include informing them and achieving wide acceptance of newly introduced circular solutions.

Recycling nutrients is the basis for closing nutrient loops (and successfully implementing the European Union (EU) Farm to Fork strategy^1^). Using locally available human excreta as a sustainable, economical resource of nutrients may be a solution for the production of bio-based fertilisers that can substitute for mineral fertilisers, and thereby promote environmentally friendly food production (Häfner et al. 2023, p. 1; Krause et al. 2021, p. 1107) and provide a circular solution. Sewage sludge has been used as a fertiliser across Europe for many decades; however, several European countries have introduced more stringent requirements (on concentrations of other heavy metals, synthetic organic compounds and microbial contamination) in comparison with the EU directive (86/278/EEC); hence, its use is limited (Hudcová et al. 2019, p. 104). Furthermore, human excreta is considered unwanted waste that creates environmental problems (Senecal and Vinnerås 2017, p. 651) that hinder the circular transition. Barriers vary, from technological (Aliahmad et al. 2023, p. 2) to legislative ambiguity, even in countries that are progressive in the subject, such as Sweden (McConville et al. 2017, p. 153). Analyses of social acceptance are rare, and results vary country by country (cf. Lienert and Tove 2009, p. 556). Urine recycling is advanced in Switzerland and Sweden, for example, and on the basis of the opinions of stakeholders, user acceptance is also good, but as soon as they have to work for it, they are no longer interested in the concept of urine recycling (Aliahmad et al. 2023, p. 9).

To provide a more in-depth understanding and to improve the use of human excreta-derived fertilisers (HEDF) and therefore help the green transition to close the loop in a wider spatial context, this study investigates the social acceptance of HEDF across three European pilot regions, revealing the spatial differences and acceptance patterns in response to the following research question: How and why does public acceptance of HEDF vary across localities, as shaped by perceived benefits and risks and contexts?

Hence, this research aims to reveal the main motivational factors underlying the social (un)acceptance (risk-benefit judgements) of the use of HEDF among stakeholders and everyday people in different spatial contexts. For this purpose, an overarching framework is proposed that nests the circular influencing model (CIM) within two mechanism families: (i) risk-benefit judgements shaped by utility-oriented adoption logic (attitudes, norms, and perceived utility) and (ii) contextual enablers (institutional trust, regulatory clarity, cultural familiarity, and proximity). To do so, we used survey questionnaires targeting a wider audience, focus group interviews and semistructured interviews of stakeholders in three case study regions (in Sweden, Germany and Spain) of the Horizon Europe P2GreeN project (grant agreement No. 101081883) and evaluated the results.

After this introduction, the remainder of the paper is structured as follows. The following chapter briefly introduces the bio-based solutions investigated in the three case study regions. After a theoretical introduction, the third chapter presents the methods and materials used. In the results and discussion sections, the findings are presented by region, while in the conclusion, we emphasise the findings and formulate recommendations for policymakers.

## The use of innovative bio-based solutions

Humans produce large amounts of urine, faeces and wastewater every year. The amount of human excreta (urine and faeces) generated per year is currently estimated at 6.34 billion tons globally (see van den Broek et al. 2024, p. 3). Human excreta contains high amounts of nitrogen and phosphorus (Winker et al. 2009, p. 4091), contributing to pollution and eutrophication. On the one hand, removing these elements is an energy-intensive process for wastewater treatment plants. On the other hand, these same elements, nitrogen, phosphorus and potassium (N-P-K), are used as fertilisers in agri-food production. More fertilisers are then manufactured to replace the nutrients removed from fields during crop harvesting (Senecal and Vinnerås 2017, p. 650).

### Historical context

The use of human excreta as a fertiliser is not novel. The collection of night soil—as a euphemism for human excrement—was practised to different extents in places where urbanisation has occurred. The collection and the use of human excreta as fertiliser is more prominent in Asia than in the western part of the globe (Szczygiel 2020, p. 1). Municipal sewerage was observed in ancient and medieval times, in the Mesopotamian Empires of Assyria, Babylonia (approximately 2500 BCE), or in the Indus civilisation in Mohenjo-daro (approximately 2550 BCE) (Gray 1940, p. 939). The use of human excrement as fertiliser has been a characteristic of traditional Chinese agriculture since the Song Dynasty (1127–1279 CE). It ensured cleanliness and hygiene in cities and was considered “top-class fertiliser” during the Ming and Qing eras (1368–1912 CE) (Du et al. 2024, p. 2). The 12th century marked the beginning of the use of human waste in agriculture in Japan, and over time, night soil became increasingly important (Szczygiel 2020, p. 2). From the mid-19th century, the growing popularity of the flush toilet led to increased volumes of water in urban faeces collectors, which considerably diluted night soil and compromised its value for fertilisation (Gandy 2004, p. 366; Kawa et al. 2019, p. 43). The adaptation of the hydraulic sanitation system aligned with emerging conceptions of hygiene and cleanliness, reshaping urban social life and governance and sparking ongoing debate and contestation worldwide (Kawa et al. 2019, pp. 43–44)^2^.

### Current trends

On the one hand, since the 1970 s, the value of human waste as fertiliser has significantly diminished due to increased use of chemical fertilisers and a growing population, leading to serious waste management issues in some parts of the world (Wang 2024, pp. 735–736). On the other hand, as sewage sludge from urban wastewater treatment plants contains organic matter and various nutrients, sludge has been used as a fertiliser across Europe for many decades. This has had positive effects on soil quality, reduced the reliance on inorganic fertilisers and promoted the circular use of nutrients. Although the application of sewage sludge to agricultural soils promotes the circular use of nutrients, it is also recognised as a significant source of secondary environmental pollution because it contains many pollutants (Giwa et al. 2023, p. 2). The amount of contaminants and associated risks may differ depending on the local characteristics and the nature of the sludge being treated and applied (Buta et al. 2021, p. 10; Pulkrabová et al. 2019, p. 2456; van den Berg et al. 2020, p. 6) 3. Concerning the EU Eurostat report, the EU livestock population is continuously declining^4^, which also means that the amount of livestock manure—even in the case of an existing optimal manure management system (cf. Oenema et al. 2007, pp. 262–263)—for potential fertilisation is also decreasing. This means that to replace N-P-K, more mineral and artificial fertilisers should be produced to maintain the linear fertilisation production line.

The Farm to Fork (F2F) strategy represents the European Green Deal’s dedicated framework for transforming the food system, designed to steer production, processing, distribution and consumption towards outcomes that are simultaneously healthier, fairer and environmentally sustainable, while keeping farm incomes viable. Launched in 2020, it set headline targets for 2030 to halve the overall use and risk of chemical pesticides, reduce nutrient losses by 50% (implying at least a 20% reduction in fertiliser use) and expand organic farming to 25% of EU agricultural land. Delivery is meant to come through a mix of legislation, standards, and research initiatives. It includes, for instance, improved soil and nutrient management and sustainable public procurement^5^. Progress has been uneven, and there have also been some setbacks (e.g., the withdrawal of the Sustainable Use of Pesticides Regulation^6^).

In line with the objectives of the European Green Deal, the work programme 2021–22 of the European Commission brought N/P flows back within safe ecological and planetary boundaries, calling for a proposal for innovation actions to explore innovative solutions to support the transfer of resources and services between rural/coastal and urban/industrial environments while limiting N/P emissions and other emissions exacerbating pollution, biodiversity loss and climate change^7^. To fulfil this requirement, the Horizon Europe P2GreeN project (Grant No. 101081883)—among other things—implements innovative practices and technologies at different governance and stakeholder levels to promote increased dialogue and collaboration, thereby encouraging behavioural change and public acceptance of recovered bio-based products as fertilisers^8^. This is important since human excreta are seen as an unwanted waste. In recent decades, several investigations have been launched (e.g., Esculier et al. 2019; Krause et al. 2021; Morée et al. 2013; Trimmer and Guest 2018; Winker et al. 2009) to draw our attention to the use of processed human excreta as a fertiliser. One of them is the aforementioned P2GreeN project, which tests bio-based, innovative solutions across different geographies and contributes to the EU goals mentioned previously.

## Description of the study areas

**Fig. 1:**
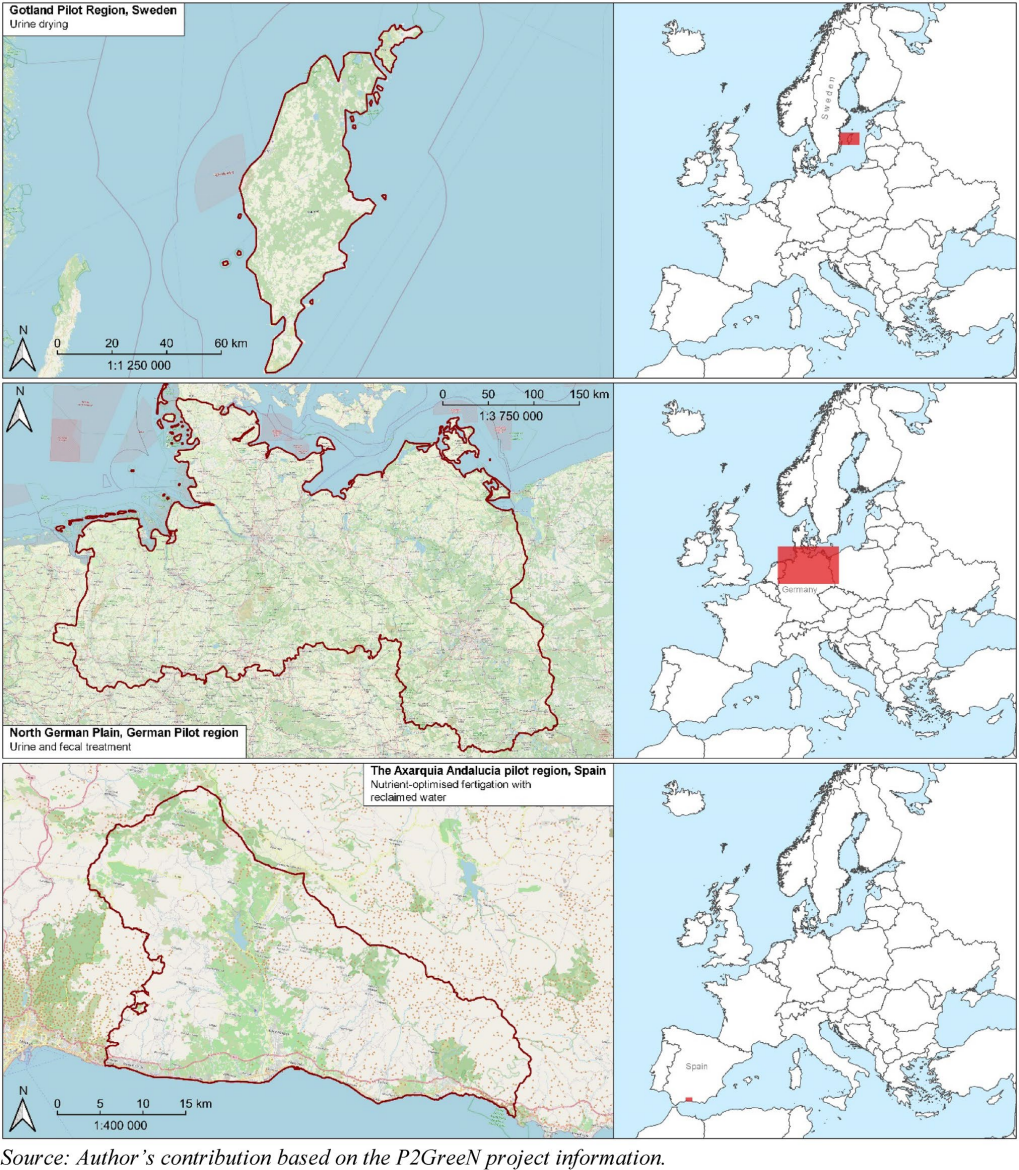
The three pilot regions and their treatments in the P2GreeN project

As stated above, the Horizon Europe P2GreeN project aims to contribute to a paradigm shift in the agri-food supply chain by shifting urban and rural areas from a linear to a circular system of resources and nutrients. To do so, solutions that focus on the circular flow of nitrogen (N) and phosphorus (P) are being developed and tested. To this end, innovative N/P recovery solutions using human excreta (faeces, urine) from urban areas and their processing into safe fertilisers that are suitable for agricultural production in three pilot regions (P2GreeN pilot regions^9^), namely, Gotland in Sweden, the North German Plain region and the Axarquia region in southern Spain, are being tested (Fig. 1). In its second phase, the feasibility of these bio-based innovative solutions will be tested in Italian, Greek and Hungarian regions.

### Gotland

In the Gotland pilot region (Sweden), urine is gathered from special urine collector toilets at festivals. After the stabilisation for nutrient retention, the urine is dried out (cf. Simha et al. 2020, p. 205), and the reclamation process is employed to produce fertiliser in pellet form. The resulting bio-fertiliser is used to fertilise and grow barley that is used by the local craft beer maker company, and the product (beer) is sold—among others—at local festivals, thereby closing the loop^10^.

### North German plain region

In the German pilot region, a source-separator toilet is used. Aurin (VunaNexus 2021) is processed from urine by hydrolysis and biological treatment, transforming ammonia into nitrate to eliminate odour; after this, micropollutants are also removed. Sanitisation and concentration are performed through distillation (cf. Häfner et al. 2023, p. 3). The faecal matter is sanitised in an aerated container, where pathogens are eliminated. In the composting process, the decomposition and recomposition of solid matter mixed with additional bulk material added by an automated auger delivery system produces a soil-nurturing fertiliser (cf. Hübner et al. 2019, p. 616; Werner et al. 2023, p. 1). The effectiveness of the fertiliser is tested by a sustainable farming operator^11^.

### Axarquia Andalusia pilot region

Water reclamation technology is tested using a smart irrigation system in the Spanish pilot region. First, treated wastewater is collected from a local wastewater treatment plant (in Algarrobo). It is further treated in a water reclamation plant utilising disc filtration and ozone disinfection to remove pathogens and contaminants. The result is a nutrient-rich effluent. Processed and reclaimed water is used to fertilise avocado and mango crops (cf. Muñoz-Sánchez et al. 2018, p. 1095).^12^

## Methods and materials

### Conceptual backgrounds

As much energy-related research emphasises, technology acceptance represents a challenge for the successful implementation of emerging technologies (e.g. Beyer et al. 2018, p. 421; Emmerich et al. 2020, p. 1). In the literature related to new technologies, “acceptability” as an attitude refers to a behavioural response either in favour of or against a technology (Emmerich et al. 2020, p. 2; Huijts et al. 2012, p. 526). The word acceptance is used in this paper with respect to the acceptance of new bio-based solutions to creating fertilisers from human excreta.

In addition to general acceptance, local resistance to technology implementation is also often referred to as the “not in my backyard” (NIMBY) phenomenon (Emmerich et al. 2020, pp. 1–2; Van Der Horst 2007, p. 2705). Given that local residents may be more concerned with personal risks (Emmerich et al. 2020, p. 2; Li et al. 2019, p. 16; Terwel et al. 2011, p. 187), this paper focuses on revealing the NIMBY phenomenon related to this often unwanted resource of fertiliser.

In risk research, it is often observed that laypeople are preoccupied with minor risks while ignoring other risks that pose significant threats (Fischer et al. 1991, p. 303). Therefore, both the questionnaire—which targeted everyday people, or laypeople—and the interviews—aimed at stakeholders—investigated worries about these new bio-based solutions derived from human excreta. Perceived risks associated with new food technologies, such as health concerns or perceived unnaturalness, can heighten neophobia. Conversely, emphasising the health benefits and sustainability of new food products can mitigate these fears (Rodrigues Romano et al. 2025, p. 1).

Another factor that determines attitudes and behaviour is trust. The term “trust” is multifaceted (Mularska-Kucharek and Brzeziński 2017, p. 84), and research on spans many disciplines, with outstanding theoretical and empirical results (Bodor et al. 2020, p. 4). On the basis of Sztompka’s definition, trust is “betting on the possible future actions of others” (Sztompka 1999, p. 25). As a subjective disposition, trust is formed because of the effect of psychologically relevant life experiences with emotional and moral contents (Bodor et al. 2020, p. 4). The social function of trust supports the assertion of the various forms of cooperation (Sztompka 1999, p. 25). People’s social trust is influenced by their perceptions of the credibility, fairness, competence and transparency of institutions (referred to as institutional trust) (Rothstein and Stolle 2008, p. 441; You 2012, p. 701; Zmerli et al. 2007, p. 35). Institutions can fill a key function in reflection on a given challenge (Bodor et al. 2020, p. 2; Gifford 2011, p. 290); hence, in our case, it is important to identify which institutions individuals trust the most to introduce and spread a safe, new bio-based fertilisation option.

The introduction of an innovation as a green transition is resource-intensive. Pro-environmental-related research on environmental quality (e.g. Vicente et al. 2021), renewable energy (e.g. Baranyai and Varjú 2017) and environmentally friendly products (e.g. Trivedi et al. 2015) often investigates the theoretical construction of “willingness to pay” (WTP) or “willingness to pay more” (WTPM) to obtain an idea about people’s intent to obtain a more sustainable environment. In addition, understanding consumers’ WTP is essential, as consumers’ demands drive the need for production (Zeng et al. 2024, p. 1345) and provide the opportunity to maximise the uptake of new solutions. Another reason is that innovations are resource-intensive in their initial stages, and to spread sustainable solutions, incentives are needed.

The concept of WTP is widely used to gauge consumer preferences and the monetary valuation of goods and services. However, there is evidence suggesting that WTP may not always reflect actual consumer behaviour. WTP can be influenced, for instance, by the retail atmosphere (Boonchai et al. 2021, pp. 484–486), past consumption habits (Wathieu 2004, pp. 587–589), and the format in which goods are presented (called the real-object advantage phenomenon) (Walsh-Snow et al. 2025, pp. 8–9). However, there is often a gap between consumers’ positive perceptions of products (e.g., organic foods) and their actual purchasing decisions. This gap, known as the intention–behaviour gap, suggests that while consumers may express a high WTP, this does not always translate into an actual purchase because there are other aspects (e.g., the origin of the food) that have greater importance (Bernabéu et al. 2022, pp. 364–366).

It is evident that the channel of communication is of fundamental importance in raising awareness and environmental consciousness. Thus, identifying space-specific information channels is key to the spread of an innovation and increases the level of acceptance.

### Analytical framework of the CIM

**Fig. 2:**
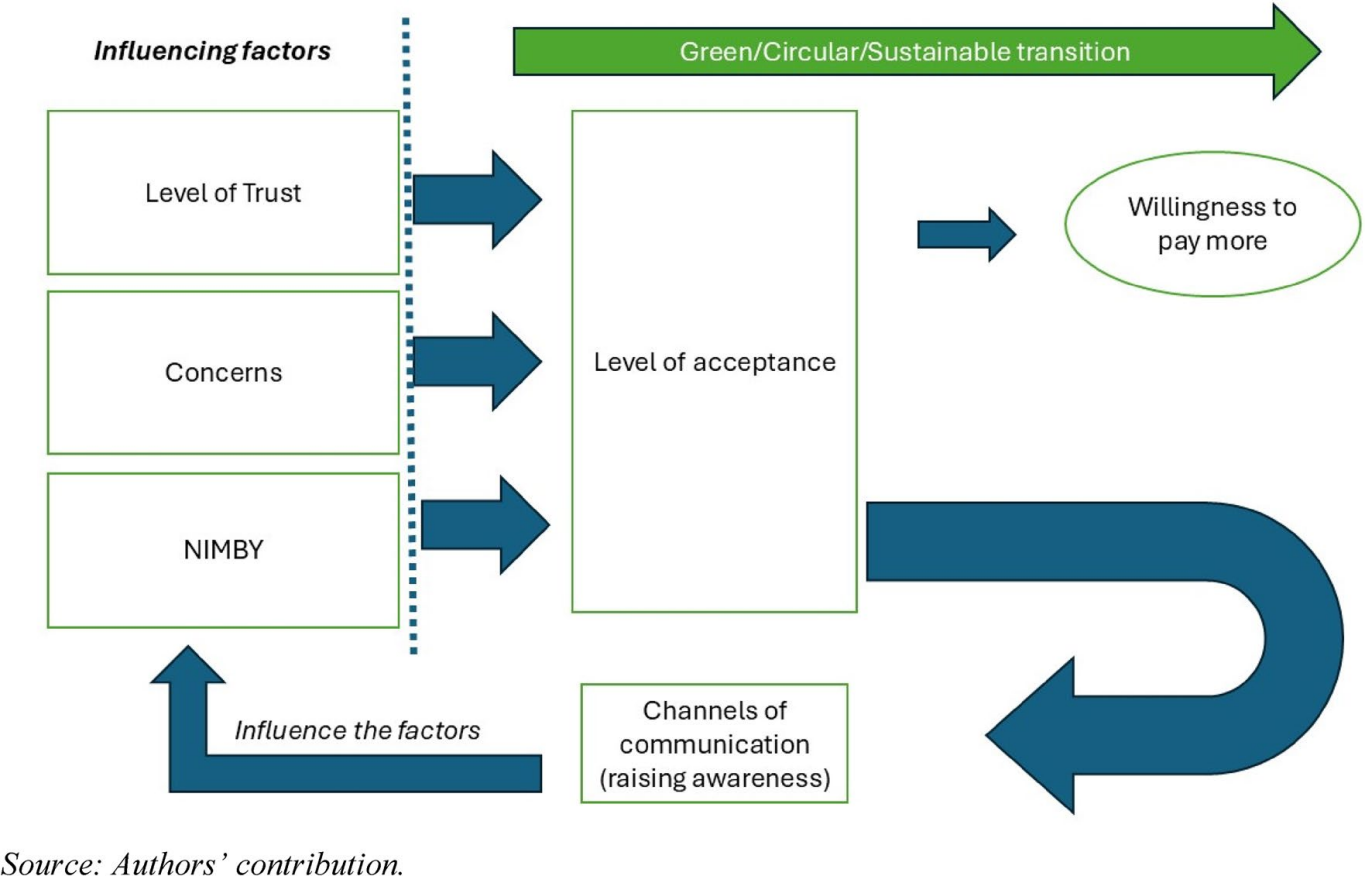
The circular influencing model (CIM) for increasing the level of acceptance

The following analytical model (Fig. 2) shows the relationships between the influencing factors and acceptance that affect willingness to pay (among other factors that are not investigated here). Key messages should be formulated based on the level of acceptance and should provide feedback to the wider public and stakeholders—via appropriate communication channels—to influence or, if possible, to increase the level of acceptance. The whole process may then promote a green, circular transition.

### Data collection

Three interrelated research methods were used. (The number of achieved persons in each of the three regions for each of the forms of data collection can be seen in Table 1.) In each pilot case, an online questionnaire was first developed, primarily to explore the attitudes of ordinary people. The questions in the online survey were created by the author, and the questions reflected the theoretical aspects mentioned above. Researchers from the case study regions were involved in the formulation of the questions to reflect the case specificity. The questionnaire (see the Annex) started with a demographic block followed by a block of questions on the importance of different social issues. In this introductory block, not only general social problems but also environmental-related challenges were subject to opinion.

After two questions on openness to innovation were answered, the next block of the survey focused on trust, looking at which stakeholders people trusted the most. Perceived behavioural control (10.i) was also detected after a question on attitude (10.h) ((cf. Ajzen 1991, p. 179) although it was not a factor this research aimed to reveal). These questions were followed by questions investigating the phenomena of NIMBY and WTP.

In the survey, the questions focused on two types of “locality” related to the NIMBY phenomenon. In one question, the respondent was asked to answer a hypothetical question about a plant near where he or she lives that produces fertiliser from human excreta. The other question hypothesised that a park near the residence would be fertilised with this type of fertiliser. A Likert scale of 1 to 10 was used for the questions measuring acceptance and evaluating the value of the new technologies. In addition to the webpage and social media pages of the P2GreeN project, the dissemination of the questionnaire was managed by the leaders in each case study region. The aim was to have at least 50 questionnaires completed in each region. The link to the English questionnaire was available on the project website and Facebook page, while the questionnaires were translated into the national languages (Spanish, Swedish, German) and distributed by the partners through their personal contacts. The questionnaires were completed between September and December 2023. A total of 54 complete responses were received from the Swedish region, 84 from the Spanish region and 249 from the German region. Given that the survey was not representative and that the distribution of the links did not reach all age groups or cover all educational backgrounds or social groups, the results were essentially used to identify the main phenomena and to draw attention to the more relevant differences between regions.

In parallel with the online survey, focus group interviews were conducted with stakeholders in each of the three pilot regions. Seven participants in each region were selected from among the participants in the experiment (separator toilet manufacturers and manure producers), potential users (farmers), researchers, and representatives of water suppliers and food processors in each region. As the cases differed, the composition of the participants differed somewhat between the regions. The focus group interviews were conducted by representatives of the consortium partners in their native language following a detailed guide. The answers to the questions were summarised, and the focus group interviews were audio-recorded; these answers were used in the present analysis.

Between January and March 2024, semistructured interviews were conducted with five additional stakeholders per region in an effort to reach experts from municipalities. The semistructured interviews, which lasted approximately half an hour and were conducted online in English, involved questions that were not asked in the focus group interviews. The interview questions, which followed a general set of questions exploring attitudes towards the environment and climate change, covered NIMBY, trust, willingness to pay for community investment, and generational differences. The interviews also reflected on the findings of the questionnaire and focus group interviews and sought to further elaborate on what was said there by the respondents.

**Table 1:** Number of people in each of the three regions for each of the forms of data collection

	Focus group interviews	Survey (questionnaire)	Semistructured interviews
Spain	7	84	5
Germany	7	249	5
Sweden	7	54	5

Both the individual interviews and the focus group interviews were analysed via content analysis, where the categories were defined by the analytical framework. Given that the amount of material to be processed was not relatively large, the analysis was conducted without special software. By identifying the factors of concern, NIMBY and trust, and the most important channels of communication, spatial patterns and space-related key messages can be formulated for policymakers.

Throughout the empirical research, the author followed the Science Ethics Code of the Hungarian Academy of Sciences (25/2010. (V. 4.)^13^, which is based on the ESF Code of Conduct and the All European Academies Code of Conduct for Research Integrity. The interviewees were asked for oral consent at the beginning of the interview, which was recorded only for research purposes, and they were assured of complete anonymity. A written consent form was signed in the case of the in-person focus group interviews, which were conducted by the region’s experts under the author’s guidance. Before completing the survey, on the first page, the respondents were informed about the project, about the use of the data collected, about the GDPR and the ethics regulations of the conducting institution and about the possibility of asking questions. It was stated that by starting the questionnaire, the respondent acknowledges and accepts that the data (the responses they provide) would be used anonymously and only for research purposes.

## Results

### Some basic figures from the surveys

A basic (descriptive statistical) analysis of the responses revealed that women are more willing to respond in each case study region. These findings may confirm observations suggesting that compared with men, women are more inclined to engage in certain types of pro-environmental behaviour than men (Hunter et al. 2004, p. 677; Xiao and McCright 2014, p. 241; Zelezny et al. 2000, p. 443). However, these results are not robust (Vicente-Molina et al. 2018, p. 95).

**Fig. 3:**
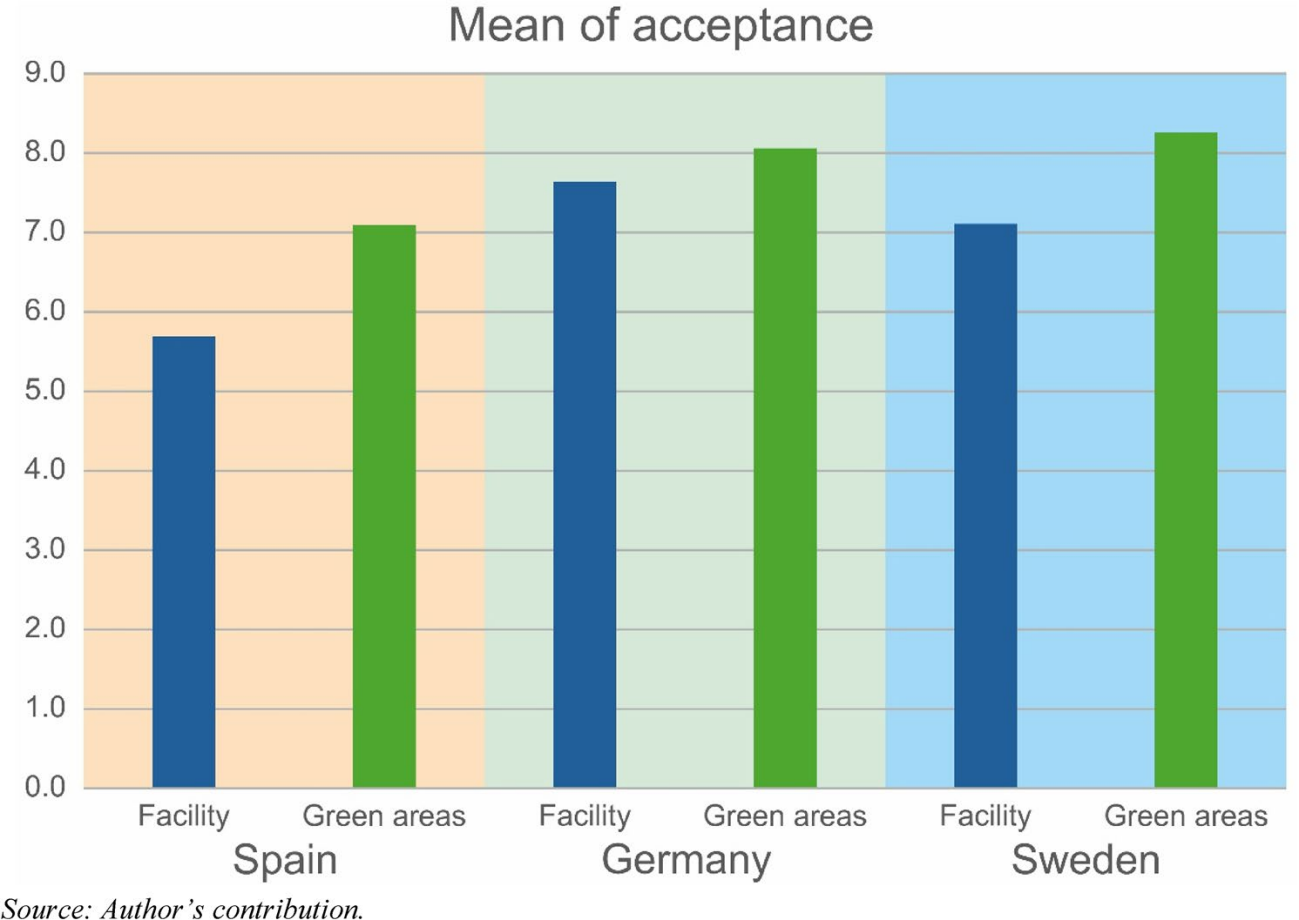
Acceptance of human excreta-based fertiliser-producing facilities and green areas fertilised by human-based fertiliser in the German, Swedish and Spanish pilot regions

To explore the NIMBY phenomenon, we asked two questions. First, the respondents were asked how they would feel if fertiliser made from human waste/sewage manure was applied to the green space near their homes and how they would feel if a factory was built near their homes to make a nutrient replacement product from sewage/human waste. The responses reveal that respondents in all three regions are more likely to accept the fertilisation of green areas than the establishment of a manure factory (Fig. 3). Furthermore, Fig. 3 shows that German respondents are the most likely to accept these actions.

**Fig. 4:**
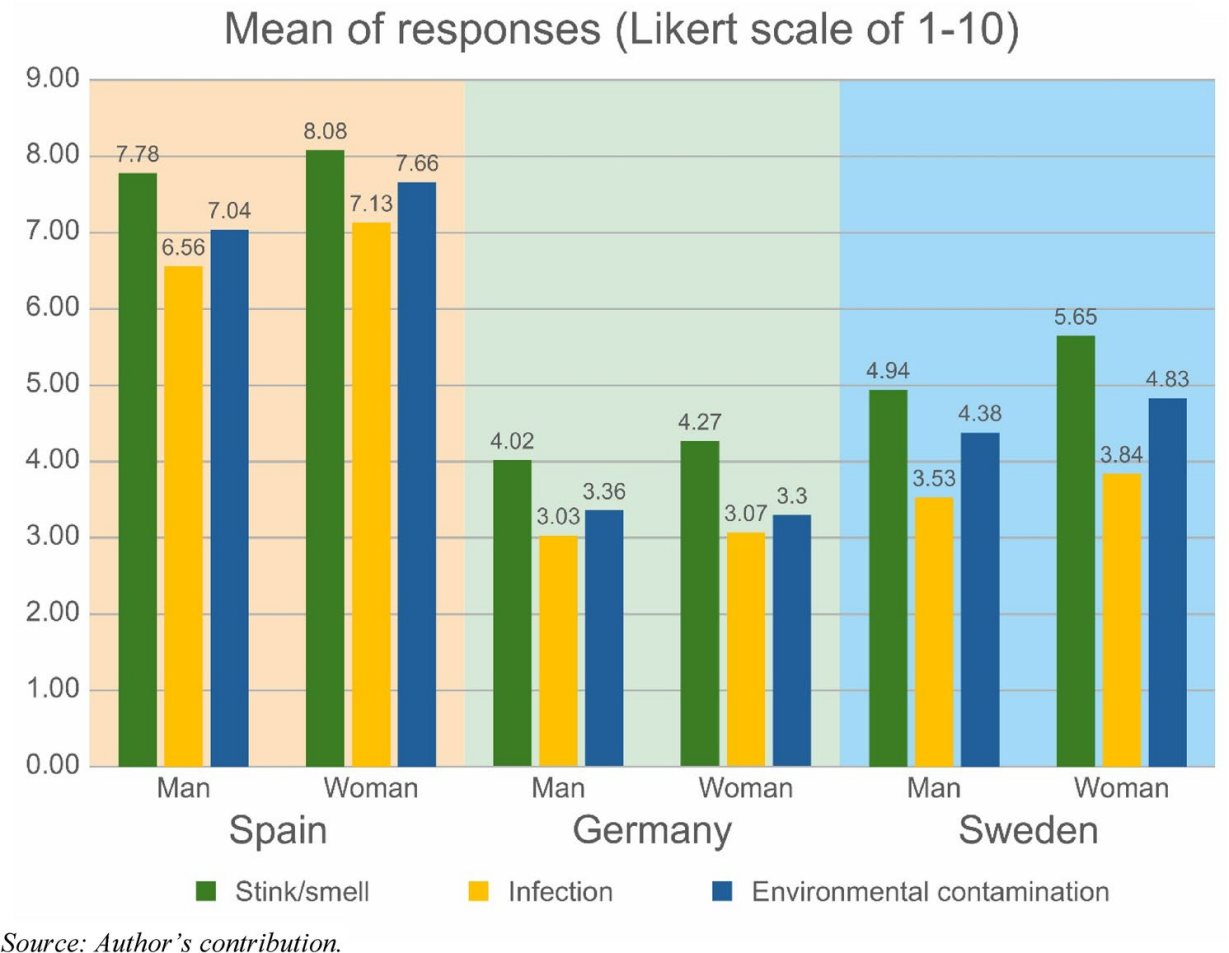
The level of concern about smell, infection and environmental contamination in the three pilot regions (mean of 1 to 10 Likert scale)

The questionnaire included three questions on fear. As shown in Fig. 4, the respondents were most afraid of the smell when using human-origin manure. However, it is also striking that Spanish respondents are almost twice as concerned about all the factors.

**Fig. 5:**
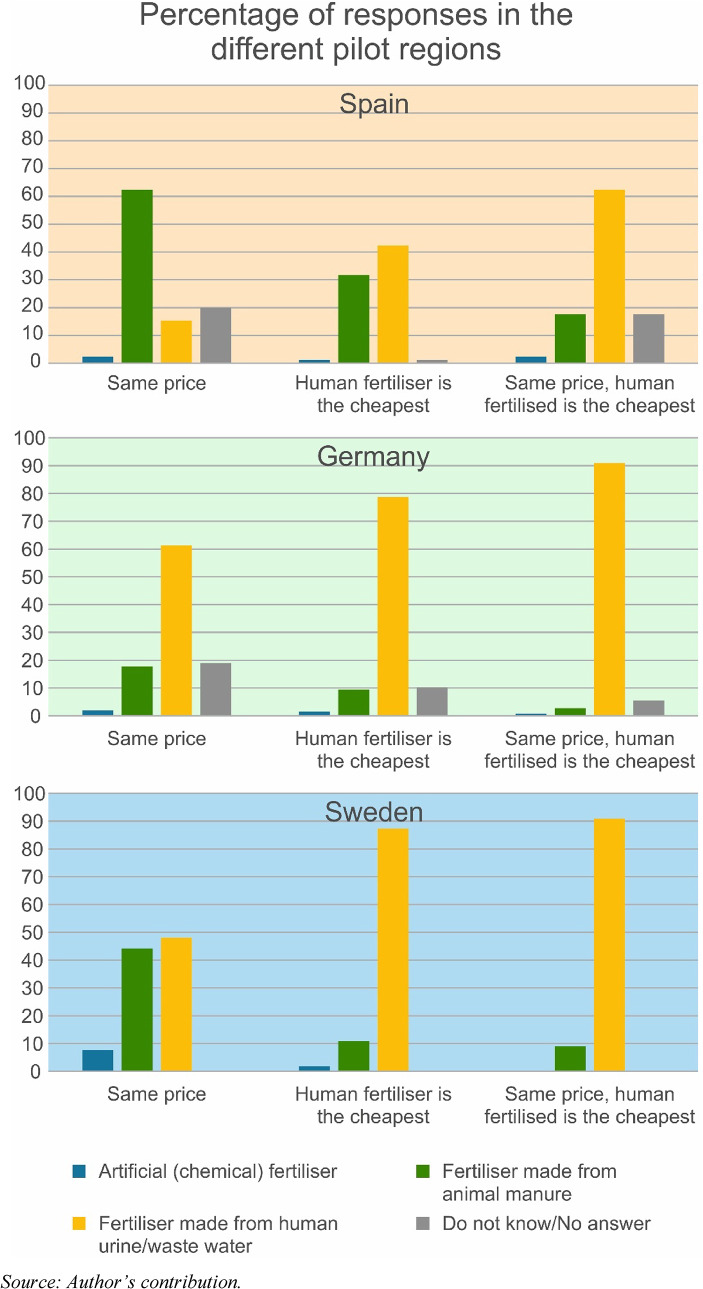
Choice between vegetables produced with different types of fertilisers in different situations (number of respondents in each sample region)

The next block of questions asked when the respondents would choose products produced with manure of human origin. Here, in addition to price, the questionnaire tested the role of the influence of health consciousness. As shown in Fig. 5, in all regions where manure-produced vegetables are less expensive, respondents are more likely to buy them, especially when it is assumed that these types of vegetables are the healthiest. This trend is in line with the analysis of Elliott et al. 2024; who reported that cost as a perceived barrier (relationship strength (RS) = 1.67) is a substantial factor, whereas health consciousness (RS = 1.82) and healthy eating intention (RS = 1.7) are high-level factors that influence healthy and sustainable diets (see details in Elliott et al. 2024, p. 33).

**Fig. 6:**
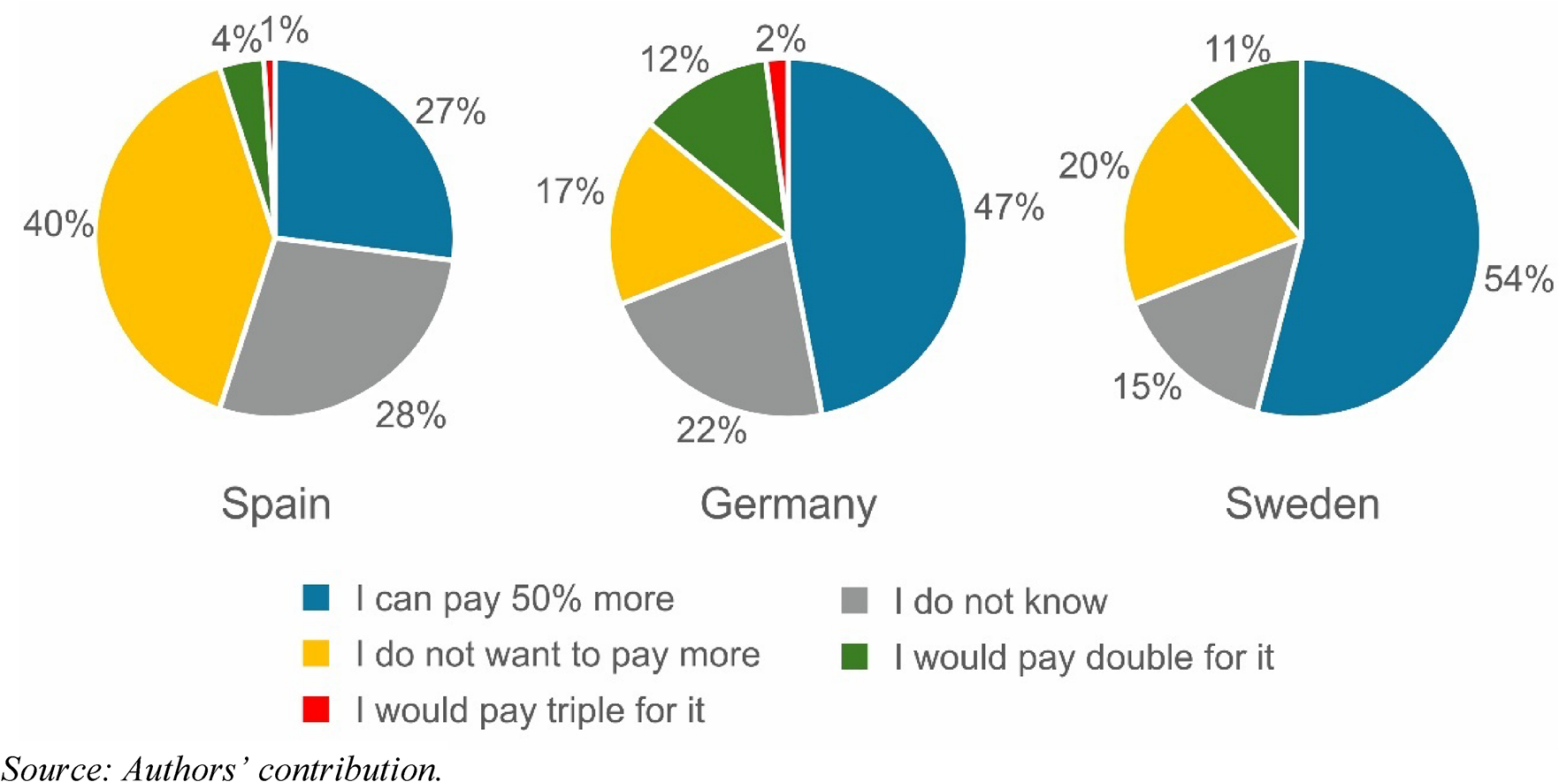
Extent of WTP for sustainably produced vegetables fertilised with manure of human origin

To assess willingness to pay, a question was designed in which the respondent had to choose between two types of vegetables. One was a cheap product fertilised with artificial fertiliser, while the other was more expensive but was fertilised in a sustainable way with environmentally friendly manure of human origin that met the criteria of circular economy principles. The question is how much more the respondent would pay for such a sustainably produced product. As shown in Fig. 6, a very small proportion of respondents would pay double or triple the price for this type of sustainable product. The proportion of undecideds (not known) is relatively high, while those who would be willing to pay 50% more for such a product are distributed differently across the regions. Proportionally, approximately twice as many people would pay more for this type of product in the Swedish and German regions than in the Spanish region, which is presumably related to the economic performance of the regions. It is important to consider that there is no uniform environmentally conscious behaviour (i.e., previous research has shown that environmentally conscious action depends on the type of action involved, e.g., environmentally friendly transport or selective waste collection) (Steg and Vlek 2009, p. 314). Additionally, the different intentions to pay more for environmental issues depend on socio-spatial and economic characteristics. For instance, according to the results of the Special Eurobarometer 550 survey, published in 2024, the WTP in the EU (for sustainable products that are easier to repair and/or can be recycled in an environmentally friendly way) was highest in Sweden, followed by Germany (7th) and Spain (10th) (European Commission 2024). In this case, although the question asked in the Eurobarometer survey was in the domain of the circular economy, the concrete intention that was asked differed. The Eurobarometer question did not quantify the ratios of WTP, but the order and magnitude of the differences between countries were similar, which can be found in the research presented in this paper. With respect to the orders and magnitude of the difference, similar results can be found in the 2011 and 2014 Eurobarometer surveys, where the question measured the willingness to pay more for environmentally friendly products (European Commission 2011, 2014). A similar gap in the proportions can be found between Sweden and Spain in a 2008 Eurobarometer survey, where the question asked about the future intention and past actions of environmentally friendly purchases (European Commission 2008). What these surveys have in common is that Sweden is ranked first in the EU in all of the surveys.

### Results of the interviews

The results of the focus group interviews and the individual semistructured interviews are presented below by case study region.

#### Axarquia Andalucía, Spain

In the Spanish region, the potential for and barriers to the use of reclaimed water for irrigation and fertilisation as a bio-based innovative solution are measured and analysed by colleagues in the Horizon Europe P2GreeN project. According to stakeholders, the use of such a new bio-based innovative solution essentially depends on the position of the person involved, i.e., the type of stakeholder. This means that in practice, farmers may accept the use of this new type of fertiliser, especially as it compensates to some extent for water scarcity in Andalusia and Spain. However, consumers are already having doubts. Although this alternative method of fertilisation is environmentally sustainable, it cannot be labelled organic because of regulations. However, any product that is not organic is generally viewed with suspicion and, as one stakeholder put it in extreme terms, criminalised.

Given this concern, stakeholders highlighted odour as potentially the most disturbing factor. In addition, stakeholders are also concerned about pharmaceutical residues. A typical hot topic is water quality across Spain. For this reason, politicians typically avoid using words such as “residue” or “waste”. Thus, in the Spanish case, policymakers typically want to avoid the term “wastewater” when describing a new bio-based innovative process, such as the one presented in this paper.

When asked about the social differences, the respondents highlighted mainly the generation gap and the digital divide that exists there, which can act as a barrier to quick access to relevant information. However, they also highlighted as a generational challenge the fact that young people today believe that they have the right to do everything; thus, they use what they want or deny what they want. This in turn can have an impact on the introduction and location of new bio-based innovative solutions.

The interviews also revealed the points that could help the new solution become widespread and popular. For example, the use of reclaimed water from wastewater protects the water basin. Or the fact that these types of solutions are similar to free-range eggs, i.e., they are produced in a sustainable way. Using these new bio-based innovative solutions aligns with the concept of a short supply chain. In addition, as has been said, this solution is not very different from the use of conventional livestock manure, which may contain residues of pharmaceuticals (as one of the interviewees explained), and yet nobody is concerned. Perhaps the most important comment was that wastewater should not be seen as a waste but as a resource.

The most credible and widely accepted sources for delivering messages, according to the opinion of the stakeholders, are the local governments/municipalities. In addition, local wastewater treatment companies also have a strong reputation. Social media and local newspapers are seen as the most important channels for delivering messages.

#### Gotland, Sweden

In the Swedish case study region, P2GreeN researchers are analysing the transformation of urine into pellets and its use as fertiliser. As one of the interviewees mentioned, the use of urine is not new in Sweden; hence, social acceptance may be greater (see also the results of the survey). Like in the Spanish region, it was mentioned that compared with food processors and consumers, farmers accept this type of new solution much more easily. Additionally, stakeholders noted that the more processed a product is, the more acceptable this type of bio-based innovative solution is. As one stakeholder noted, this may also be because in Sweden, young people no longer know how and from what a meal and its ingredients are made. Additionally, the importance of separating the concepts of fertilisation and the use of pesticides when information is disseminated to a wider audience was highlighted, as these two concepts are not necessarily clear to everyday people. With respect to stakeholders’ concerns, the main concern of the interviewees was pharmaceutical residue.

One of the key points in favour of this new solution could also be, in this case, the local availability of this “recycled” material. Moreover, some form of sustainability certificate may also help to promote the wider availability of such products that are fertilised with urine. In the Swedish case as well, the interviewees noted the similarity of the fertiliser to conventional farmyard manure. According to the interviewees, a sewage tax could also help to promote this new solution.

The Swedish stakeholders believe that the companies involved play the primary role in informing the public in a transparent way about investments in new solutions. In addition, local governments can play an important role in promoting the emergence of alternative fertilisation solutions.

Two further important channels for the dissemination of solutions were also mentioned: social media and local newspapers.

#### The German case

Stakeholders involved in the German trial typically had far fewer doubts about products fertilised with manure from human origin. The main argument was that this type of nutrient supplementation is similar to classical animal-based fertilisation. Generalised trust was also considered to be high, which was supported by one interviewee saying that if something appears in the supermarket, it is trustworthy, and the customer assumes that it has been checked. However, according to another interviewee, this type of product should not be marketed because society is not ready for it. Moreover, very few people would be curious about how the product was made or how it was farmed. It is also worth clarifying that we are talking about an agricultural process, not the product itself, in regard to alternative fertilisation techniques. In addition, organic products are also fertilised^14^. The question is whether this needs to be communicated to the consumer. In the discussion in the focus group interview, the consensus emerged that in the case of a farm shop, it should be, but in a supermarket, not necessarily.

With respect to concerns, German stakeholders are most worried about hormone residues.

If possible, German stakeholders would also promote products fertilised with this type of manure as organic. Additionally, an important aspect and important message is that the use of dry toilets or compost toilets (Roach et al. 2015) is a form of water savings, which is also a hot topic in Germany, as one of the interviewees expressed.

In the German case, municipalities are also seen as being the most credible partners in the dissemination of new solutions. In addition, NGOs play an important monitoring role, as they are also more responsive.

Moreover, here social media and local newspapers play the most important role in the spread of information related to new solutions, according to the interviewees.

## Discussion

### Risk-benefit judgement (concerns, willingness to pay and NIMBY)

A range of barriers, psychological, social, and practical, currently limit the public’s willingness to embrace fertilisers derived from human excreta. One key barrier is the psychological “fear of the unfamiliar”. Consumers may instinctively be repulsed by the idea of eating food supplemented with human waste, a reaction rooted in perceived impurity or danger. Gustavsen et al. (2025) demonstrated that food neophobia can significantly decrease the level of acceptance. In a Norwegian survey, individuals with higher neophobic tendencies were less willing to pay for lettuce grown with fertilisers made from fish sludge or human waste, preferring conventional produce and even demanding a price discount for unconventional produce (Gustavsen et al. 2025, p. 6). In our case, in this respect, a spatial difference from north to south can be identified, with the level of WTP decreasing. This also correlates quite well with a recent survey by Eurostat on the attitudes of Europeans towards the environment^15^.

Psychological and cultural barriers often translate into heightened public concern about health risks. For example, our Spanish respondents emphasised strong fears about odour, pathogens, and environmental contamination from human fertiliser use. Such concerns echo the findings in the UK, where health and safety concerns led to lower acceptance of human-derived fertiliser for food crops (Pickering et al. 2024, p. 18). Furthermore, research from outside Europe (and from the U.S.) also supports that there is notable consumer prejudice and farmer hesitance towards using HEDFs. This is partly due to the potential presence of contaminants and the general discomfort or disgust associated with human waste (Schreiber et al. 2021, p. 229; van den Broek et al. 2024, pp. 10–12).

Our survey revealed that respondents across all three countries were more willing to accept the use of human-derived fertiliser on distant green spaces (e.g., public parks or remote fields) than to accept a processing facility or fields using such fertiliser in their immediate vicinity. This pattern indicates conditional support, as people like the idea of recycling waste into fertiliser; however, they prefer it to happen out of sight and away from their daily lives. Pickering et al. (2024) reported that participants in an English survey were far more comfortable with the use of human-sourced fertilisers in public parks than with the food they personally consumed (Pickering et al. 2024, p. 18). This finding is in line with the work of Segrè Cohen and colleagues (2020b) in the U.S., where acceptance of human urine-derived fertilisers is higher for nonedible plants because of concerns about pharmaceutical residues and environmental risks (Segrè Cohen et al. 2020b, p. 5301). Such NIMBY tendencies reflect underlying risk perceptions and a desire to avoid any potential nuisances (odours, traffic, stigma) near one’s community. The high acceptance observed in Germany may partly be due to a lower NIMBY sentiment, which is possibly a result of greater trust in how local authorities manage such projects, or “environmental pragmatism”, where benefits are seen to outweigh inconveniences.

Van den Broek and colleagues (2024) reported that compared with conventional inorganic fertilisers, properly treated human-derived fertilisers do not present greater pathogen risk and that overall, they can be used safely when appropriately matched to crops and soils. This finding indicates that many health concerns are more perceived than evidence-based, given that traditional manure and synthetic fertilisers also carry risks (pathogens in manure and environmental pollution from excess nutrients in synthetics). Nonetheless, the need for robust quality control remains a barrier, with continuous monitoring and research being needed to ensure that emerging contaminants (such as pharmaceutical metabolites) are kept at safe levels (van den Broek et al. 2024). Until such assurances are effectively communicated to the public and codified in regulations, technical uncertainties continue to hinder full social acceptance.

### Cultural context and locality

As seen in the results, social acceptance of human-derived fertilisers is highly context-dependent and varies significantly across different localities. In the Swedish case, where environmental consciousness is strongly embedded in the culture, acceptance was notably higher, with Germany also showing relatively high openness, whereas the Spanish region exhibited the most scepticism towards using fertilisers from human excreta. These territorial differences align with broader patterns: regions that prioritise environmental sustainability and have prior familiarity with nutrient recycling practices tend to be more receptive. For example, Swedish stakeholders noted a longstanding familiarity with urine-based fertilisation and the presence of sustainability certifications, which together have helped normalise the idea of recycling human nutrients in agriculture. In contrast, Spanish participants showed less openness, influenced by a lack of such traditions and a more cautious public discourse around water and waste.

These results are consistent with those of cross-cultural research indicating that the historical and cultural context shapes attitudes towards human-sourced fertilisers (Pickering et al. 2024, p. 18), which revealed strong differences between the UK (England) and Japan: Japanese respondents, who come from a culture with a long history of night soil use, were significantly more accepting of human excreta-based fertiliser for food production and expressed fewer health concerns, whereas English respondents were more wary of food applications but were more comfortable with using these fertilisers on non-food green spaces (Pickering et al. 2024, p. 18). Notably, English respondents were more open to fertiliser from human waste being used in public parks than to that from crops, reflecting a degree of removal in regard to food supply (Pickering et al. 2024, p. 18), which interferes with our results. This reflects the long history and territorial differences (Asia vs. Western countries) in the use of human excreta as fertiliser (cf. Szczygiel 2020, p. 1). Similarly, in Ghana, while fresh human faeces are viewed negatively, dried or treated faeces are more acceptable as fertilisers because of reduced visual intimidation and symbolic transformation (Buit and Jansen 2016, p. 97).

### Communication and engagement strategies

Overcoming the above barriers will require proactive communication and stakeholder engagement strategies tailored to address public concerns and local contexts. A recurring theme in both our findings and the literature is that how information about human-based fertilisers is conveyed can make a decisive difference in social acceptance (Segrè Cohen et al. 2020b, p. 5300). Effective communication should aim to demystify these fertiliser products, emphasise their benefits, and directly address safety concerns. One strategy is to highlight the environmental and economic benefits in a way that resonates with the target audience’s values. Gustavsen et al. (2025) stress that messaging should focus on food safety, environmental sustainability, and the benefits of nutrient recycling to mitigate neophobic reactions and build public support (Gustavsen et al. 2025, p. 6). In our study, many respondents indicated that they would accept human-derived fertilisers if they believed that the end products (e.g., vegetables) were healthier or more cost-effective. This suggests an opening for communication campaigns to frame these fertilisers as part of producing healthful, sustainable food, for example, by noting that they enable organic cultivation without synthetic chemicals or that they conserve resources in a circular economy model. Importantly, communicators should avoid terminology that inadvertently triggers disgust.

As noted, Spanish communities prefer the term “reclaimed water” or similar euphemisms over any mention of “waste”. Adopting positive or neutral terminology, such as “eco-fertilisers” or “recycled nutrients”, and focusing on the purification and treatment steps can help reframe the narrative from one of waste and risk to one of innovation and resourcefulness. Indeed, van den Broek et al. (2024) argued that public outreach and education are essential for human-based fertiliser feasibility, to inform consumers and farmers about the multifaceted benefits and safety of these products after proper processing (van den Broek et al. 2024, pp. 13–14). Such outreach could include public demonstrations (e.g., community garden projects using urine-based fertiliser) or information campaigns that share scientific evidence (for instance, that no increase in pathogen risk has been found compared with that associated with conventional fertilisers). Another promising approach is the use of certification and quality labels to build trust. In Sweden, the presence of sustainability certifications was noted to bolster confidence in urine-fertilised crops. The development of an official label or certification for fertilisers derived from human excreta, one that guarantees that they meet safety and quality standards, could alleviate consumer worries. For example, a new certification such as *“Local*,* Circular*,* Sustainable (LCS)”* has been proposed to explicitly acknowledge fertilisers of human origin as analogous to accepted organic ones, thereby reassuring consumers that these products are controlled and sustainable. Such branding could normalise the product’s image and allow producers to market environmental advantages in a credible way. In our case, the role of videos was not at the forefront of solutions used to increase acceptance. The use of videos was not mentioned in the focus groups or in the individual interviews. However, the results of studies in the U.S. emphasise that videos, compared with other communication strategies, may be more effective at addressing consumer interests around how food is produced (Segrè Cohen et al. 2020a, p. 9). This may be a helpful solution in the Swedish case for the younger generation to increase food consciousness. As Segrè Cohen et al. (2020a) argue, “this may be even more pertinent to a novel food or food-related product, such as HUDF^16^” (Segrè Cohen et al. 2020a, p. 9).

Equally important is the choice of messengers and the building of trust through stakeholder engagement. Our findings showed that local authorities and municipalities are seen as highly credible actors for promoting new circular solutions. In the German case, in particular, the respondents and interviewees indicated that they trusted municipal bodies to oversee and communicate innovations such as human-based fertiliser more so than private companies did. Involving local government early, for example, through city council-led pilot projects or public forums, can lend institutional legitimacy to the initiative and ensure that information is transparent and tailored to local concerns. Partnering with trusted institutions to co-host informational sessions or to validate the safety of the fertiliser can further increase public confidence. Additionally, engagement should be two-way: providing channels for community members to voice questions and concerns helps address misconceptions and shows responsiveness. Participation by NGOs can also be harnessed positively; rather than having NGOs only in critical monitoring roles, they can be invited to participate in project design and oversight, which may reassure sceptics that an impartial party is keeping an eye on risks (this was reflected in our interviews, where NGOs were noted as important monitors). In this P2GreeN project, a field visit was also used to show and better understand the process of how fertilisers are created. Additionally, in stakeholder engagement workshops, producers presented and showed the process with supporting videos. These actions are in line with the research of Farias and colleagues (2025), who emphasised that conferences with field visits and farmer panels can bridge the gap between research and practical application (Farias et al. 2025, pp. 18–20) and hence help better understand the process and the real risks and benefits.

On the media front, leveraging local media and social networks is vital for widely disseminating information. Our study revealed that social media and local newspapers were among the most important channels for the spread of news about fertiliser innovations in all three regions. Crafting compelling local news stories, for instance, highlighting a successful harvest that used human-based fertiliser or a positive testimonial from a respected local farmer, can help normalise the concept in the public eye. By using familiar and trusted communication avenues and by engaging stakeholders as partners rather than passive recipients, it is possible to gradually develop social acceptance from the ground up. These findings are in line, for instance, with two studies conducted in China, where the empirical results demonstrated that the size and improvement of the social network positively affect farmers’ cognition of a new type of fertiliser (in this case, controlled-release fertiliser) or fertiliser use efficiency and that communication intensity with neighbouring farmers positively affects adoption behaviour (Ma and Yang 2023, pp. 13–14, Chang et al. 2023, p. 101854).

## Conclusion and policy implications

This project analysed the social acceptance of bio-based innovative solutions implemented in the Horizon Europe P2GreeN project. In support of the EU Green Deal’s transition to a circular economy and contributing to the Farm to Fork strategy, the feasibility of using manure from human origin in three regions was investigated. In the Swedish region of Gotland, pellets are made from urine and used for barley cultivation in the trial. In the German region, urine and faeces are collected separately using a separator toilet, where the urine is used to produce Aurin^®^ and the faeces are composted. In the Spanish region of Axarquia, an experiment is being carried out in which reclaimed water is used as nutrient replenishment and irrigation water.

Social acceptability was examined on the basis of the circular influencing model (CIM), where, in addition to the phenomenon of NIMBY, concern was also considered to be a barrier. The levels of trust in the different actors and institutions and the possible communication/information channels were also examined. In addition, the role of willingness to pay to support sustainable solutions was analysed.

Not surprisingly, social acceptance varies across nations and regions. Essentially, it is higher in regions that are generally more positive about the environment, where people see environmental consciousness as a very important issue. The Swedish region stands out in this respect, but the German region also has a higher acceptance of new bio-based innovative solutions. Among the three regions investigated, the Spanish region is the least open to the use of bio-based innovative solutions based on human nutrient replenishment.

The survey results reveal a consistent trend of greater acceptance of the application of human excreta-based fertilisers to green spaces than the establishment of organic waste processing facilities close to residential areas. This reflects a degree of openness to the concept of nutrient recycling, albeit tempered by the not-in-my-backyard (NIMBY) phenomenon. Notably, compared with their Spanish and Swedish counterparts, German respondents demonstrated the highest levels of acceptance, suggesting a greater degree of environmental pragmatism.

Concerns regarding odour, infection, and environmental contamination were prevalent, with Spanish respondents expressing significantly higher levels of apprehension. This heightened concern may stem from cultural and sociopolitical factors, including the sensitive discourse surrounding water quality and the avoidance of terminology linked to waste. These findings underscore the importance of addressing sensory and health-related concerns as part of public engagement strategies.

The study also reveals that economic incentives and health consciousness significantly influence consumer behaviour. The respondents demonstrated a clear willingness to purchase vegetables fertilised with human-derived manure when the products were perceived as healthier or more cost-effective. However, the willingness to pay a premium for such products varies by region, with respondents from Sweden and Germany being more inclined to pay higher prices than those from Spain. This aligns with broader patterns of environmental engagement and economic capacity observed in previous Eurobarometer surveys.

Interviews with stakeholders provided further insights into the nuanced dynamics shaping acceptance and implementation. In Spain, the generational divide and regulatory constraints limiting the classification of such products as organic emerged as critical barriers. Conversely, Swedish stakeholders emphasised their familiarity with urine-based fertilisation and the role of sustainability certifications in fostering acceptance. In Germany, high levels of generalised trust and alignment with traditional agricultural practices facilitated broader acceptance, although concerns about hormone residues persisted.

Across all regions, the role of local governments and municipalities was consistently highlighted as crucial for disseminating information and fostering trust. Additionally, social media and local newspapers emerged as vital channels for public engagement. These findings underscore the necessity of regionally tailored communication strategies that address specific cultural, economic, and regulatory contexts.

Although evidence comes from three European regions, the mechanisms identified, institutional trust, contamination concerns, neophobia, and fit-for-place communication, are not region-bound. The emphasised communication and engagement practices offer transferable levers for circular nutrient initiatives in other socioecological contexts.

The uptake of this new bio-based innovative solution is also hampered by the fact that there is currently no legal possibility to label products produced with this type of fertiliser as organic. One solution could be the introduction of a new certificate: “Local, Circular, Sustainable” (LCS) (highlighting the similarity of fertiliser of human origin to that of animal origin, the use of which is accepted).

Overall, this research contributes to the growing body of knowledge on circular economy practices by elucidating the conditions under which innovative fertilisation methods may gain public acceptance. By addressing identified barriers and leveraging region-specific facilitators, policymakers and stakeholders can enhance the adoption of sustainable nutrient recycling practices, ultimately contributing to the advancement of more resilient and resource-efficient agricultural systems.

## Limitations and potential future research

This study has several limitations. First, the empirical evidence stems from three European regions with distinct cultural and regulatory contexts, which constrains external validity. The results should therefore be interpreted as context contingent rather than universally generalisable. Second, the data are cross-sectional and rely on self-reports; stated attitudes, acceptance and willingness-to-pay (WTP) may not translate into revealed behaviour in real purchasing or adoption settings, and responses may be affected by social desirability or momentary salience (e.g., media coverage). Third, the sampling frames for the public survey and stakeholder interviews were non-probability and, for some subgroups, modest in size, which can introduce selection bias and limit the statistical power for interaction effects (e.g., by age, education or profession). Fourth, the category of “human excreta-derived fertilisers (HEDF)” comprises heterogeneous technological options (e.g., processed urine products versus composts/derivatives). Our measures did not fully disaggregate perceptions by technology pathway, crop type or treatment standard, potentially masking within-category differences. Finally, the circular influencing model (CIM) is used here as an analytical lens rather than a fully specified causal model; we cannot make strong causal claims about pathways between communication, psychological drivers and acceptance. Future work should address these limitations. In addition, future research should focus more on the causality of communication channels and the increase in social acceptance of these new solutions.

## Supplementary Information

Below is the link to the electronic supplementary material.

Supplementary material 1 (DOCX 44.0 kb)

## Data Availability

The recording and transcription will be deposited at the KRTK Databank: https://adatbank.krtk.mta.hu/after the closure of the project. Data are also deposited in the P2GreeN project data storage system following its data management plan. Datasets will also be stored at the Innovation Platform of the P2GreeN project.

## Footnotes

1
https://food.ec.europa.eu/horizontal-topics/farm-fork-strategy_en.

2 An example from the UK on the difficulties of transport planning is presented in the University of Nottingham blog by Hannah O’Regan: https://blogs.nottingham.ac.uk/classicsandarchaeology/2020/06/10/frozen-charlotte-and-the-night-soil/.

3
https://www.eea.europa.eu/en/european-zero-pollution-dashboards/indicators/long-term-impacts-of-sludge-spreading-on-agricultural-land#footnote-7JoPU.

4
https://ec.europa.eu/eurostat/en/web/products-eurostat-news/w/ddn-20240521-2.

5
https://food.ec.europa.eu/horizontal-topics/farm-fork-strategy_en; https://food.ec.europa.eu/system/files/2020-05/f2f_action-plan_2020_strategy-info_en.pdf; https://www.europarl.europa.eu/factsheets/en/sheet/293547/the-farm-to-fork-strategy.

6
https://food.ec.europa.eu/plants/pesticides/sustainable-use-pesticides_en.

7
https://ec.europa.eu/info/funding-tenders/opportunities/portal/screen/opportunities/topic-details/horizon-cl6-2022-zeropollution-01-02;callCode=null;%20freeTextSearchKeyword=nitrogen;%20matchWholeText=true;%20typeCodes=0,1,2;statusCodes=31094501,31094502,31094503;programmePeriod=null;%20programCcm2Id=null;%20programDivisionCode=null;%20focusAreaCode=null;%20destination=null;%20mission=null;%20geographicalZonesCode=null;%20programmeDivisionProspect=null;%20startDateLte=null;%20startDateGte=null;%20crossCuttingPriorityCode=null;%20cpvCode=null;%20performanceOfDelivery=null;%20sortQuery=sortStatus;%20onlyTenders=false;%20topicListKey=topicSearchTablePageState.

8 For more information, please visit the Horizon Europe project’s website: https://p2green.eu/.

9
https://p2green.eu/pilot-regions/.

10
https://p2green.eu/pilot-regions/.

11
https://p2green.eu/pilot-regions/.

12
https://p2green.eu/pilot-regions/.

13
https://mta.hu/data/dokumentumok/bolyai_osztondij/tudomanyetikai_kodex_kgy_20100504.pdf.

14 It is important to note that products fertilised with manure of human origin cannot be certified as being organic at present, as this type of fertilisation is not covered by the certification scheme.

15
https://europa.eu/eurobarometer/surveys/detail/3173.

16 Human urine-derived fertilizer.
