# Supplementary material for: From fork to farm, locally: social acceptance pathways for human excreta-derived fertilisers across three European regions
Source: Socioecol Pract Res. 2025 Dec 9;7(4):419–37. doi: 10.1007/s42532-025-00236-x (PMC12727824; doi:10.1007/s42532-025-00236-x)
Supplement: Supplementary file 1 — Supplementary material 1 (DOCX 44.0 kb) [file 42532_2025_236_MOESM1_ESM.docx]

ANNEX

The questionnaire

**Social acceptance – Survey**

Linked to the EU's Green Deal and the idea of a Circular Economy, the main aim of the [P2GreeN project](https://p2green.eu/) is to support innovations that turn human sanitary waste into fertiliser.

At the same time, we believe that innovation and the circular economy can only be effective if we take into account society's attitude and acceptance of innovation. The questionnaire seeks to answer this.

Within the survey, you will be asked for some personal data. Responses will be anonymised.

Should you have any questions about this Call for Evidence, or the SSH CENTRE project more broadly, please contact Viktor Varjú (varju.viktor@krtk.hu, survey supervisor).

Starting the survey, I understand and accept that a minimum of personal data (gender, age, occupation, social status) are recorded by the host server at the premises of the P2GreeN partner and protected by the General Data Protection Regulation (GDPR). They cannot be used for purposes other than the one for which they were gathered (understanding social acceptance). They are for the exclusive use of their recipient (KRTK) and are subject to confidentiality. (Data protection guidelines can be found here: <https://adatbank.krtk.mta.hu/en/adatvedelem/>)


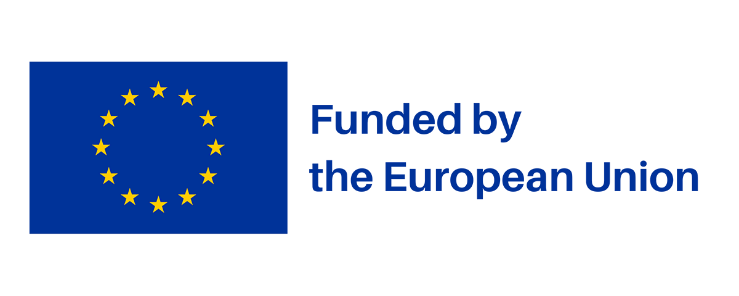


‘European Union’s Horizon Europe research and innovation programme under grant agreement No° 101081883

1. Please indicate the year of your birth:

2. Please, indicate your gender:

Woman

Man

Non-binary

Prefer to self-describe:…………….

Prefer not to say

3. What is your nationality?

….

4. In which country do you currently live?

…

5. What is your highest level of education? (Choose one.)

a. primary school

b. secondary school

c. Bachelor's degree

d. Masters’ degree

e. PhD/DLA

f. Other, please specify:……

h. No answer

6. How would you describe your profession? (Choose one.)

a. student

b. employed

c. self-employed

d. unemployed

e. retired

f. apprentice

g. Other, please specify…

h. No answer

7. What kind of work do you do?

1. intellectual, degree required

2. intellectual, non-degree required

3. physical, skilled

4. manual, skilled worker

5. manual, unskilled worker

6. farmer

7. self-employed trader, service provider

8. self-employed producer, non-agricultural

9. Other, please specify:…

8. Which of the following best describes the financial situation of your household?

1. we live without financial problems and we can save substantial money

2. we can manage our monthly income well and we can save some money as well

3. our income just covers our expenses

4. we have financial problems from month to month

5. we live in poverty

0. does not wish to answer

9. On a scale of 1 to 10, please rate how threatening you think the following social problems are (1 = not threatening at all, 10 = very threatening. You can also use a value in between)

1. Healthcare 1-2-3-4-5-6-7-8-9-10 0(KN/NA)

2. Armed conflicts 1-2-3-4-5-6-7-8-9-10 0(KN/NA)

3. Pandemics 1-2-3-4-5-6-7-8-9-10 0(KN/NA)

4. Inflation 1-2-3-4-5-6-7-8-9-10 0(KN/NA)

5. climate change 1-2-3-4-5-6-7-8-9-10 0(KN/NA)

6. migration 1-2-3-4-5-6-7-8-9-10 0(KN/NA)

7. unemployment 1-2-3-4-5-6-7-8-9-10 0(KN/NA)

8. education 1-2-3-4-5-6-7-8-9-10 0(KN/NA)

9. poverty, hunger 1-2-3-4-5-6-7-8-9-10 0(KN/NA)

10. terrorism 1-2-3-4-5-6-7-8-9-10 0(KN/NA)

11. overpopulation 1-2-3-4-5-6-7-8-9-10 0(KN/NA)

12. water scarcity 1-2-3-4-5-6-7-8-9-10 0(KN/NA)

13. environmental pollution 1-2-3-4-5-6-7-8-9-10 0(KN/NA)

10. How much do you agree with the following. Please rate the statements from 1 to 10, where 1=strongly disagree and 10=strongly agree. You can also use a value in between.

10.a) I often read about the latest technologies

1-2-3-4-5-6-7-8-9-10 0(KN/NA)

10.b) I usually use the latest technologies (e.g. in mobile phone; machines) in my life

1-2-3-4-5-6-7-8-9-10 0(KN/NA)

10.c) I trust that companies in the private sector ensure that safe facilities are built.

1-2-3-4-5-6-7-8-9-10 0(KN/NA)

10.d) I trust that companies in the private sector operate this system safely.

1-2-3-4-5-6-7-8-9-10 0(KN/NA)

10.e) I trust that the government or the responsible state authorities adequately consider the needs of local residents.

1-2-3-4-5-6-7-8-9-10 0(KN/NA)

10.f) I trust that the government or the responsible state authorities make a responsible decision whether a new technology can be introduced or not.

1-2-3-4-5-6-7-8-9-10 0(KN/NA)

10.g) I trust that the government or the responsible state authorities will carry out safety checks to ensure safe operation.

1-2-3-4-5-6-7-8-9-10 0(KN/NA)

10. h) The environment is important to me.

1-2-3-4-5-6-7-8-9-10 0(KN/NA)

10.i) My lifestyle is perceived by my friends as environmentally aware.

1-2-3-4-5-6-7-8-9-10 0(KN/NA)

11. Imagine that a facility that creates fertilizers from human urine/excrement or waste water would be built next to your place of residence. What kind of feelings does that evoke in you? 1-10 scale; 1 = very negative feelings disagree, 10 = very positive feelings

1-2-3-4-5-6-7-8-9-10 0(KN/NA)

12. Imagine, that the field/park/green area next to your place of residence is fertilised by a fertiliser transformed from human urine/excrement or reclaimed water from waste water.

What kind of feelings does that evoke in you? 1-10 scale; 1 = very negative feelings disagree, 10 = very positive feelings

1-2-3-4-5-6-7-8-9-10 0(KN/NA)

12.a: What do you feel exactly?

…

12.b: How much do you worry about the followings? (Please rate your worry from 1 to 10, where 1=you are not worried about it at all; and 10= you are very much worried. You can also use a value in between.)

Worry about the stink/smell 1-2-3-4-5-6-7-8-9-10 0(KN/NA)

Worry about the infection 1-2-3-4-5-6-7-8-9-10 0(KN/NA)

Worry about the environmental contamination of the soil/water 1-2-3-4-5-6-7-8-9-10 0(KN/NA)

13. Imagine that you can buy three types of vegetables in a market. One is fertilized by a.) artificial fertiliser, one is fertilised with b.) fertiliser made from animal manure, and the third is fertilized with c.) fertiliser made from human urine/waste water. Which would you choose:

13.a) If all three vegetables have the same price. The one fertilised by:

a.) artificial fertiliser,

b.) fertiliser made from animal manure

c.) fertiliser made from human urine/waste water

0- KN/NA

13.b) If the cheapest vegetable is the one that was fertilised by fertiliser made from human urine/waste water:

a.) artificial fertiliser,

b.) fertiliser made from animal manure

c.) fertiliser made from human urine/waste water

0- KN/NA

13.c) If all three vegetables have the same price and the one that was fertilised by fertiliser made from human urine/waste water seems to be the healthiest:

a.) artificial fertiliser,

b.) fertiliser made from animal manure

c.) fertiliser made from human urine/waste water

0- KN/NA

13.d) Imagine, that there are two types of vegetables on the market. One is fertilised with cheap artificial fertiliser, the other (more expensive) with fertiliser made from human urine/waste water. The latter, however, is 100% environmentally friendly, sustainable and follows the principles of a circular economy. How much more would you be willing to pay for this vegetable? (Please choose one.)

a) I do not want to pay more

b) I can pay 50% more

c) I would pay double for it

d) I would pay triple for it

e) I do not know

14. In case you would like to receive information about the results of this survey, please write here to your e-mail address:
